# Supplementary material for: Validation and characterisation of a DNA methylation alcohol biomarker across the life course
Source: Clin Epigenetics. 2019 Nov 27;11:163. doi: 10.1186/s13148-019-0753-7 (PMC6880546; doi:10.1186/s13148-019-0753-7)
Supplement: Supplementary file 1 — Additional file 1. R2 between DNAm-Alcs and measures of alcohol intake (log(g/day +1)) and AUDIT score, in ARIES parents at midlife and offspring at adolescence with adjustment of DNAm-Alcs for concurrent smoking. [file 13148_2019_753_MOESM1_ESM.pdf]

		N	R^2			
			5 CpGs	23 CpGs	78 CpGs	144 CpGs
Midlife						
	Alcohol intake	1 049	3.84	4.32	6.7	7.57
	AUDIT	1 013	5.04	5.73	8.85	9.00
Adolescence						
	Alcohol intake	626	0.12	0.26	0.57	0.61
	AUDIT	620	0.00	0.05	0.66	0.88

Additional File 1. R^2 between DNAm-Alcs and measures of alcohol intake (log(g/day + 1)) and AUDIT score, in ARIES parents at midlife and offspring at adolescence with *adjustment of DNAm-Alcs for concurrent smoking*.
