# Supplementary material for: Validation and characterisation of a DNA methylation alcohol biomarker across the life course
Source: Clin Epigenetics. 2019 Nov 27;11:163. doi: 10.1186/s13148-019-0753-7 (PMC6880546; doi:10.1186/s13148-019-0753-7)
Supplement: Supplementary file 2 — Additional file 2. R2 between DNAm-Alcs and alcohol intake (log(g/day +1)) in ARIES parents at midlife and offspring at adolescence, excluding self-reported non-drinkers. [file 13148_2019_753_MOESM2_ESM.pdf]

		N	R^2			
			5 CpGs	23 CpGs	78 CpGs	144 CpGs
Midlife						
	Alcohol intake	988	3.80	4.28	6.89	7.58
	AUDIT	952	5.51	6.27	10.07	9.77
Adolescence						
	Alcohol intake	586	0.13	0.35	0.57	0.64
	AUDIT	580	0.00	0.07	0.77	1.05

Additional File 2. R^2 between DNAm-Alcs and alcohol intake (log(g/day + 1)) in ARIES parents at midlife and offspring at adolescence, *excluding self-reported non-drinkers*.
