# Supplementary material for: Validation and characterisation of a DNA methylation alcohol biomarker across the life course
Source: Clin Epigenetics. 2019 Nov 27;11:163. doi: 10.1186/s13148-019-0753-7 (PMC6880546; doi:10.1186/s13148-019-0753-7)
Supplement: Supplementary file 3 — Additional file 3. R2 between DNAm-Alcs and alcohol intake (log(g/day +1)) and AUDIT score in HN5000. [file 13148_2019_753_MOESM3_ESM.pdf]

		N	R^2			
			5 CpGs	23 CpGs	78 CpGs	144 CpGs
HN5000						
	Alcohol intake	281	8.88	7.34	12.52	14.34

Additional File 3. R^2 between DNAm-Alcs and alcohol intake (log(g/day + 1)) and AUDIT score in HN5000.
