# Supplementary material for: Validation and characterisation of a DNA methylation alcohol biomarker across the life course
Source: Clin Epigenetics. 2019 Nov 27;11:163. doi: 10.1186/s13148-019-0753-7 (PMC6880546; doi:10.1186/s13148-019-0753-7)
Supplement: Supplementary file 4 — Additional file 4. Adjusted R2 from models regressing measures of alcohol use, alcohol intake (g/d), in ARIES parents at midlife and offspring at adolescence on the methylation β-values of CpGs used in constructing DNAm-Alcs, as was the modelling approach presented in Liu et al. 2016. [file 13148_2019_753_MOESM4_ESM.pdf]

		N	Adjusted R^2* ~ Age + Sex + BMI	+ 5 CpGs	+ 23 CpGs	+ 78 CpGs	+ 144 CpGs
Midlife							
	Alcohol intake	1 049	4.78	8.77	9.73	12.69	17.25
Adolescence							
	Alcohol intake	626	0.00	-0.63	-0.17	-2.63	-0.45

Additional File 4. Adjusted R^2 from models regressing measures of alcohol use, alcohol intake (g/d), in ARIES parents at midlife and offspring at adolescence on the methylation β-values of CpGs used in constructing DNAm-Alcs, as was the modelling approach presented in Liu et al. 2016. *See Methods for details.
