# Supplementary material for: Validation and characterisation of a DNA methylation alcohol biomarker across the life course
Source: Clin Epigenetics. 2019 Nov 27;11:163. doi: 10.1186/s13148-019-0753-7 (PMC6880546; doi:10.1186/s13148-019-0753-7)
Supplement: Supplementary file 5 — Additional file 5. Area under the curve for prediction of ‘at risk’ category of drinkers by DNAm-Alcs. [file 13148_2019_753_MOESM5_ESM.pdf]

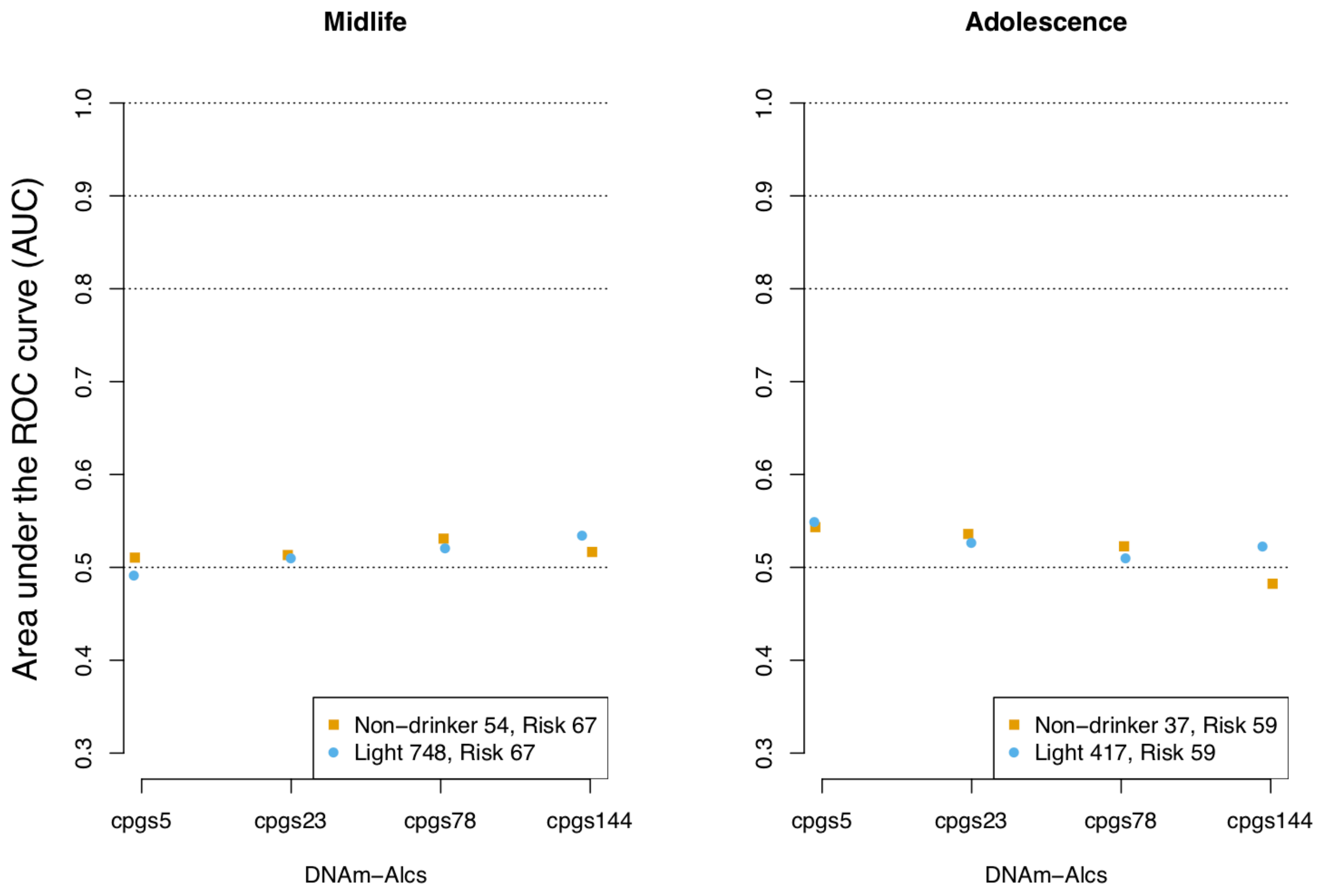

Additional File 5. Area under the curve for prediction of 'at risk' category of drinkers by DNAm-Alcs. ROC analysis was performed to discriminate at risk category of alcohol intake versus non-drinkers and light drinkers in ARIES parents at midlife (left figure) and children at 17 years of age (right figure). 'At risk drinkers' were participants who consumed 14 < g/day < 28 in women and 28 < g/day < 42 in men (N = 67 at midlife, N = 59 at adolescence); 'non-drinkers' consumed 0 g per day (N = 54 at midlife, N = 37 at adolescence); 'light drinkers' consume 0 < g per day ≤ 28 in men and 0< g per day ≤ 14 in women (N = 748 at midlife, N = 417 at adolescence).

Abbreviations: DNAm-Alcs, DNA methylation alcohol biomarkers; ARIES, Accessible Resource for Integrated Epigenomics Studies.
