# Supplementary material for: Validation and characterisation of a DNA methylation alcohol biomarker across the life course
Source: Clin Epigenetics. 2019 Nov 27;11:163. doi: 10.1186/s13148-019-0753-7 (PMC6880546; doi:10.1186/s13148-019-0753-7)
Supplement: Supplementary file 6 — Additional file 6. Area under the curve for prediction of binary alcohol categories by DNAm-Alcs in HN5000. [file 13148_2019_753_MOESM6_ESM.pdf]

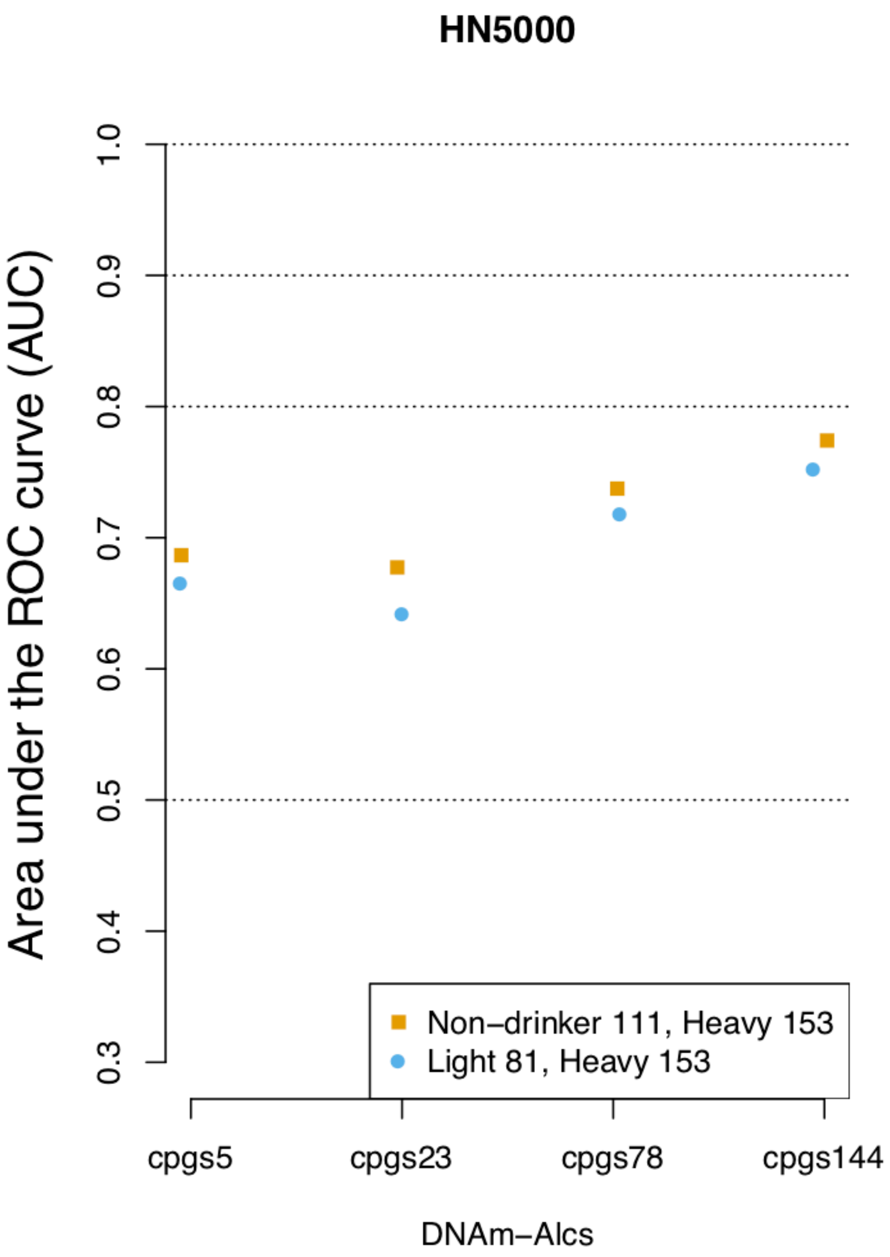

Additional File 6. Area under the curve for prediction of binary alcohol categories by DNAm-Alcs in HN5000. ROC analysis was performed to discriminate categories of alcohol intake (heavy versus non-drinkers and heavy versus light drinkers) HN5000 participants. ‘Heavy drinkers’ were participants who consumed ≥ 42 g per day in men and ≥ 28 g per day in women (N = 153); ‘non-drinkers’ consumed 0 g per day (N = 111); ‘light drinkers’ consume 0 < g per day ≤ 28 in men and 0< g per day ≤ 14 in women (N =81). Abbreviations: DNAm-Alcs, DNA methylation alcohol biomarkers; HN5000: Head and Neck 5000 clinical cohort study
