# Supplementary material for: Validation and characterisation of a DNA methylation alcohol biomarker across the life course
Source: Clin Epigenetics. 2019 Nov 27;11:163. doi: 10.1186/s13148-019-0753-7 (PMC6880546; doi:10.1186/s13148-019-0753-7)
Supplement: Supplementary file 7 — Additional file 7. Estimates of the effects of DNAm-Alc in ARIES offspring at birth and in mothers during pregnancy on offspring AUDIT at adolescence considered both in separate single-predictor models and simultaneously. [file 13148_2019_753_MOESM7_ESM.pdf]

Dependent Variable	Independent Variable(s) (DNAm Alc score)	N	β	SE	P-value	Adjusted R^2*
AUDIT at adolescence		380				
	~ Birth		0.70	0.40	0.08	0.55
	~ Pregnancy		0.46	0.43	0.29	0.04
	~ Birth +		0.67	0.40	0.10	0.49
	Pregnancy		0.38	0.43	0.38	

Additional File 7. Estimates of the effects of DNAm -Alc in ARIES offspring at birth and in mothers during pregnancy on offspring AUDIT at adolescence considered both in separate single-predictor models and simultaneously.
